# Long-read sequencing reveals the complex splicing profile of the psychiatric risk gene *CACNA1C* in human brain

**DOI:** 10.1038/s41380-019-0583-1

**Published:** 2019-11-06

**Authors:** Michael B. Clark, Tomasz Wrzesinski, Aintzane B. Garcia, Nicola A. L. Hall, Joel E. Kleinman, Thomas Hyde, Daniel R. Weinberger, Paul J. Harrison, Wilfried Haerty, Elizabeth M. Tunbridge

**Affiliations:** 1 Department of Psychiatry, University of Oxford, Oxford, UK; 2 Centre for Stem Cell Systems, Department of Anatomy and Neuroscience, The University of Melbourne, Melbourne, VIC Australia; 3 The Earlham Institute, Norwich, UK; 4 The Lieber Institute for Brain Development, Baltimore, MD USA; 5 Oxford Health NHS Foundation Trust, Oxford, UK

**Keywords:** Molecular biology, Neuroscience

## Abstract

RNA splicing is a key mechanism linking genetic variation with psychiatric disorders. Splicing profiles are particularly diverse in brain and difficult to accurately identify and quantify. We developed a new approach to address this challenge, combining long-range PCR and nanopore sequencing with a novel bioinformatics pipeline. We identify the full-length coding transcripts of *CACNA1C* in human brain. *CACNA1C* is a psychiatric risk gene that encodes the voltage-gated calcium channel Ca_V_1.2. We show that *CACNA1C*’s transcript profile is substantially more complex than appreciated, identifying 38 novel exons and 241 novel transcripts. Importantly, many of the novel variants are abundant, and predicted to encode channels with altered function. The splicing profile varies between brain regions, especially in cerebellum. We demonstrate that human transcript diversity (and thereby protein isoform diversity) remains under-characterised, and provide a feasible and cost-effective methodology to address this. A detailed understanding of isoform diversity will be essential for the translation of psychiatric genomic findings into pathophysiological insights and novel psychopharmacological targets.

## Introduction

Genomic studies have identified numerous common single nucleotide polymorphisms (SNPs) that are robustly associated with psychiatric disorders; the challenge is now to understand the underlying pathophysiological mechanisms [1]. Most of these SNPs are non-coding [2–4], implying that they mediate disease associations by influencing aspects of RNA expression. Of the various mechanisms of RNA regulation, the possibility that psychiatric risk SNPs might influence RNA splicing is particularly appealing, given its exquisite, cell type-specific regulation and its key role in determining neuronal properties [5]. Consistent with this hypothesis, at a global level, cellular studies emphasise RNA splicing as a key mechanism mediating the effect of disease-associated, non-coding variants in complex disorders, including schizophrenia [6]. Similarly, in human brain, genomic regions associated with schizophrenia are enriched for genes that show differential isoform usage across neurodevelopment [7], implicating many schizophrenia-associated risk loci in the regulation of the expression of specific RNA transcripts. Consistent with these findings, examples of associations between psychiatric risk-associated loci and the abundance of novel, alternatively spliced transcripts are beginning to emerge [8–10], complementing numerous reports of altered splicing patterns in the brains of psychiatric cases, compared with controls [11–13].

Splicing varies extensively across human development and aging [7], and between tissues. The brain exhibits one of the highest levels of splicing diversity and prominent use of tissue-specific exons, microexons and splicing factors [14–18]. However, despite extensive efforts to improve the annotation of the human genome [19], the alternative splicing patterns of many genes remain poorly described, especially in brain, and novel coding exons (many of which are enriched within brain-expressed genes [20, 21]) and transcripts are continually being discovered. This is due to the limited availability of high-quality, post-mortem human tissue and to technical limitations associated with standard approaches: most rely on short-read RNA-Seq and the reconstruction of fragmented sequences making the disambiguation of full-length transcripts difficult, particularly for large and complex genes. Benchmarking studies have shown that short-read RNA-Seq methodologies are unable to accurately reconstruct and quantitate the majority of transcript and protein isoforms [22, 23].

**Fig. 1:**
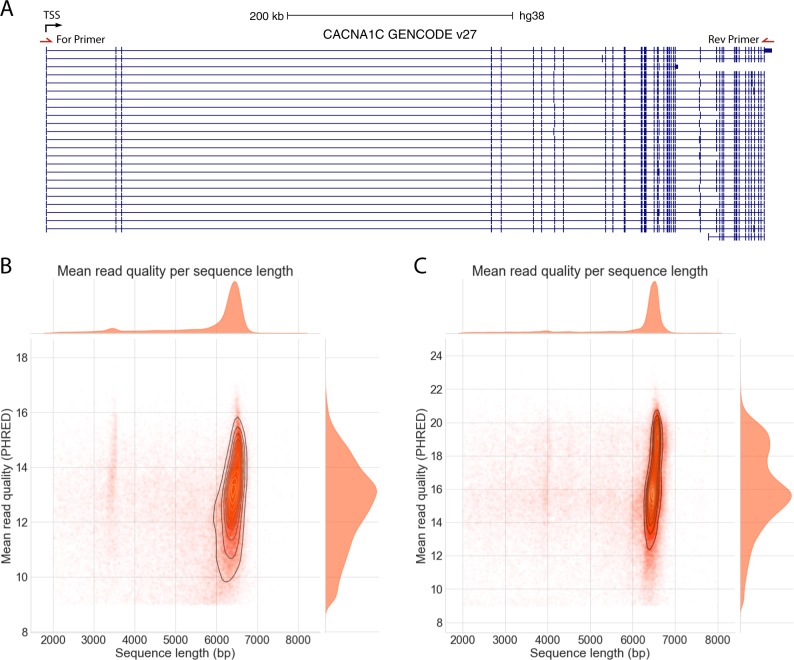
Amplicon sequencing of CACNA1C. **a** UCSC genome browser screenshot of CACNA1C isoforms annotated in GENCODE V27. All transcripts in “Basic” annotated set shown. Black arrow shows direction of transcription. TSS transcription start site. Position of forward and reverse long-range PCR primers in first exon and universal portion of final exon shown. Length vs Quality of all 2D pass reads from **b** Run 1 **c** Run 2. Most reads are the full-length CACNA1C CDS. The presence of the 3.5 kb positive control CDS spike-in can be seen in Run1. Visualisation limited to reads between 2 and 8 kb, encompassing >98% of pass reads in each run

The relative lack of knowledge about the splicing of individual human genes is typified by *CACNA1C*. *CACNA1C* encodes the Ca_V_1.2 voltage-gated calcium channel (VGCC) alpha_1_ subunit and is a leading genomically informed candidate gene for multiple psychiatric disorders [2, 3, 24, 25]. However, it is not known how its expression and/or splicing is altered in illness, or in association with genetic risk for it [26]. The *CACNA1C* gene is large (cDNA ~13433 nucleotides [nt], 2209 amino acids [aa]) and complex, with at least 50 annotated exons and 31 predicted transcripts (Gencode 27; Fig. 1a) [17]. Its size and complexity make accurate transcript identification and quantification by standard gene expression measures extremely difficult; consequently, the true full-length protein sequence of isoforms remains unclear. Information on the transcript diversity of *human* neuronal VGCC subunits is sparse: most studies have focused on rodents [18], or human cardiac tissue [27, 28]; the sole study examining VGCC splicing in human brain used a single adult sample [29]. This information is significant, both in terms of understanding pathophysiological mechanisms and for the development of novel pharmacological agents, because *CACNA1C* encodes multiple alternatively spliced transcripts, which result in functionally and pharmacologically distinct channels [30, 31].

To address these unknowns, we developed a straightforward and cost-effective approach using a long-read sequencing technology [32–34]. We combined long-range RT-PCR with long-read nanopore sequencing and a novel bioinformatics pipeline to characterise full-length *CACNA1C* coding sequences (CDSs) in post-mortem human brain. We show that the transcript structure of human *CACNA1C* is substantially more complex than currently appreciated, with numerous novel transcripts and isoforms containing unannotated exons, novel splicing junctions, and in-frame deletions that are predicted to alter the protein sequence and function.

## Methods

### Sample preparation and sequencing

Post-mortem brain tissue from three adult donors in the Lieber Institute for Brain Development repository was obtained from the Maryland Medical Examiner’s Office [35]. Subjects with evidence of neuropathology, drug use (other than alcohol), or psychiatric illness were excluded. Demographic information is presented in Supplementary Table 1.

Full methodological details are presented in the Supplementary Information. Briefly, RNA was extracted from cerebellum, striatum, dorsolateral prefrontal cortex [DLPFC], cingulate cortex, occipital cortex and parietal cortex and reverse transcribed. We selected these brain regions to investigate transcript diversity in cortical and subcortical regions known to be transcriptionally and functionally diverse [36] and to investigate to what extent the *CACNA1C* transcript profile differs between cortical regions. Full-length CDSs (from the first to last exon [focussing on ‘Exon1B’ the start exon most abundant in brain [30]]) were amplified using PCR, and barcoded and sequenced (across two runs; six samples common to both runs) using Oxford Nanopore Technology’s MinION.

### Data analysis and code availability

Our novel data analysis pipeline is available from: https://github.com/twrzes/TAQLoRe. 2D pass reads (i.e. those with a *q*-score of ≥9) with an identified barcode were mapped to the transcriptome (hg38, ENSEMBL v82) using a HPC version of BLAT (pblat-cluster v0.3, available from http://icebert.github.io/pblat-cluster/ [37]) and to the *CACNA1C* metagene containing known exonic sequences using GMAP version 2017–04–24 [38]. Reads mapping uniquely to *CACNA1C*, and with at least 50% of their length mapped were retained for further analysis.

To identify potential novel exons, we mined the alignments of the reads to the transcriptome for inserts of at least nine nucleotides in length located at the junctions between annotated exons. To further characterize candidate novel exons, we mapped the corresponding reads to the genome using LAST v926 [39]. We retained candidate exons located within the expected intronic sequences, and at least six nucleotides away from existing exons. Novel exonic sequences were subsequently introduced to the *CACNA1C* metagene (concatenation of all exons) and reads were mapped again to this new model using GMAP to enable further characterization of alternative splicing and quantification of transcript expression.

To characterize novel exon junctions, we mapped all the reads to the human genome (hg38) using LAST v926 [39]. We identified all exon junctions that showed perfect and contiguous mapping (no sequencing errors nor insertion/deletions) of the read to the genome and used canonical acceptor and donor splice sites. We then used this comprehensive set of exon junctions to correct reads with sequencing errors at mapping breakpoints by selecting the closest canonical splice site to the breakpoint.

To annotate transcripts that include novel exons and/or junctions, we parsed the CIGAR string from the alignment to the metagene, to identify the combination of exon junctions supported by each read. We subsequently clustered splicing patterns to annotate a unique set of isoforms for *CACNA1C* and enable their expression quantification. We applied a similar approach to annotate the transcripts containing novel splice sites. We filtered exons and transcripts of low abundance (i.e. supported only by one read), and clustered transcripts together to call only their longest possible variants.

Transcript expression both on exon and splice site levels was quantified using the number of reads supporting the transcript model. Reads mapping to multiple transcripts were down-weighted according to the number of transcripts they could be mapped to. Read counts were normalized across libraries using the trimmed Mean of M-values normalization method (TMM) [40]. For visualisation, all normalized counts were log_10_-transformed. Expression heatmaps and PCA plots were generated using R statistical language [41], by heatmap3 [42] and ggplot2 libraries [43], respectively. To normalise for the difference in sequencing depth between samples, we downsampled all libraries to match that with the smallest sequencing depth and recomputed transcript expression patterns.

### Quality control

A subset of novel exons and novel exon junctions found in *CACNA1C* by nanopore sequencing (Supplementary Table 2) were confirmed by PCR targeting the novel sequence followed by Sanger sequencing (See Supplementary Information for details).

RNA extracted post-mortem shows variable degradation, and its quality is usually assessed using the RNA integrity number (RIN). In order to identify the minimum RIN required for reliable amplification of the full-length CDS of *CACNA1C*, RNA samples were artificially degraded (Supplementary Fig. 1). *CACNA1C* CDS amplification in these artificially degraded samples was assessed alongside that from striatal RNA samples of varying RINs from three adult donors (Supplementary Table 3).

## Results

We successfully amplified and sequenced the full length ~6.5 kb *CACNA1C* CDS (encompassing the full intron chain of *CACNA1C*) from all samples (Fig. 1a). A minimum RIN of 6 is required, and a RIN of >7 is optimal, for amplification of full-length *CACNA1C* (Supplementary Fig. 1). Sequencing run 1 produced 112024 reads, including 52994 (47%) 2D pass reads with an identified barcode. Run 2 produced 126314 reads, including 83221 (64%) 2D pass reads with an identified barcode (Supplementary Tables 4 and 5). Most pass reads were full length, with the updated flowcell used in Run2 providing higher quality reads with a lower error rate (Fig. 1). All analyses were performed using the 2D pass barcoded reads, hereafter referred to simply as “reads”.

### Long-range amplicon sequencing reveals many novel CACNA1C exons and isoforms

We developed a bespoke alignment and mapping pipeline to maximise the transcript information obtained from nanopore sequencing reads, including the identification of novel exons, acceptor and donor splice sites, and splice junctions (Supplementary Fig. 2). Because of *CACNA1C’s* complexity, we used two complementary approaches to identify transcripts: exon-level and splice-site-level analyses. These combined approaches identified a total of 251 unique *CACNA1C* transcript isoforms present in human brain, 241 of which are novel, including the use of novel exons, novel splice sites and junctions. The identity of these transcripts can be viewed on the UCSC Genome Browser: https://genome-euro.ucsc.edu/cgi-bin/hgTracks?hubUrl=https://opendata.earlham.ac.uk/opendata/data/CACNA1C_isoforms/hub.txt&genome=hg38.

#### Exon-level analysis

**Fig. 2:**
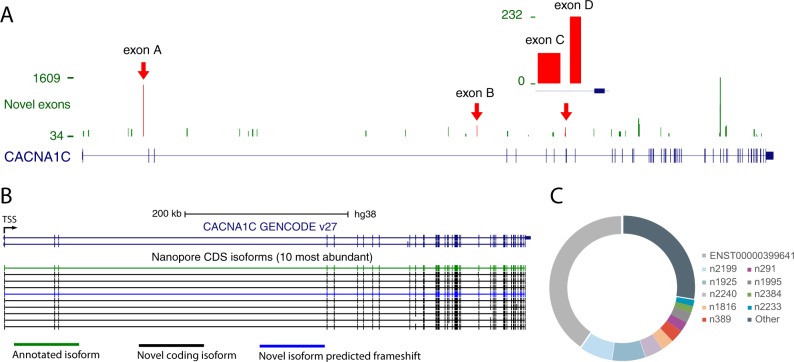
**a** Annotation and read count for novel exonic sequences within CACNA1C. Red arrows indicate exons that have been validated (Supplementary Table 2). **b**, **c** Top 10 most abundant CACNA1C isoforms identified in brain using exon-level approach. **b** UCSC genome browser screenshot of top isoforms. Colours denote transcript type. **c** Proportion of high-confidence transcripts reads from the ten most abundant transcripts

We annotated a total of 39 potential novel exons within the *CACNA1C* locus, of which 38 were identified in at least 2 individuals or tissues and supported by at least 5 nanopore reads in each library (Fig. 2a; Supplementary Data Table 1).

We validated four out of four of the novel exons (selected across the range of abundance) by PCR and Sanger sequencing by confirming the splice junctions between the novel exon and its surrounding annotated exons. We also discovered a fifth novel exon that was spliced in between the targeted novel exon and the nearest known exon. This successful validation of a selection of novel exons provides high confidence that the novel exons identified by nanopore sequencing are real and actively incorporated into *CACNA1C* transcripts.

Integration of these 38 novel exons and the mapping of the reads to the metagene enabled the identification of novel and known (i.e. annotated) transcripts. Novel transcripts incorporate novel exons, and/or novel junctions between annotated exons and/or new combinations of known junctions. To limit the impact of sequencing errors on transcript annotations, we filtered the identified transcripts retaining only those found in at least 2 libraries with a minimum of 24 reads in total (see Supplementary Data Files 2 and 3 for transcript identity and abundance, respectively). We also created a high-confidence set of transcripts with an increased minimum threshold of 100 reads (see Supplementary Data Files 4 and 5 for transcript identity and abundance, respectively). Unless otherwise stated all analyses were performed on the high-confidence transcripts set. We identified 90 high-confidence *CACNA1C* transcripts across the 6 brain regions, including 7 previously annotated (GENCODE v27) and 83 novel (Supplementary Fig. 3). Seven of the novel high-confidence transcripts contained novel exons (1.8% of high-confidence reads in total), while the remaining 76 included previously undescribed junctions and junction combinations.

As expected the most highly expressed transcript was previously annotated (ENST00000399641) and is supported by 40.2% (23.7–50%) of reads on average. In comparison, the most highly expressed novel transcript (*CACNA1C n2199*) represents on average 7.3% (2.7–25.3%) of all reads (Fig. 2b, c). Nine of the top ten expressed transcripts were novel, of which eight are predicted to maintain the *CACNA1C* reading frame, suggesting a number of these most abundant novel transcripts encode functionally distinct protein isoforms (Fig. 2b, c). Without any thresholding, 75.5% of all the reads supported previously unannotated transcripts. These results suggest that novel *CACNA1C* transcripts are abundantly expressed as well as highly numerous, and that current annotations are missing many of the most abundant *CACNA1C* transcripts. Without filtering, we found evidence for the expression of only 18 of the current set of 31 annotated transcripts (GENCODE v27).

#### Splice-site-level analysis

The exon-level transcript identification approach described above provides a robust and conservative means to identify novel exons and to characterise full-length transcript structure. However, it is relatively insensitive to small-scale variation (of the order of a few amino acids). Therefore, we also implemented a splice junction-level analysis to annotate novel splice sites and junctions in the *CACNA1C* gene model. We used a conservative approach, which relies on the identification of junctions supported by error-free mapping at the junction, and the presence of canonical splice sites. We thereby identified 497 novel splice sites, of which 393 were supported by at least 10 reads. Compared with previously annotated splice sites, novel donor and acceptor splice sites are less used (Supplementary Fig. 4). Including these splice sites, and after filtering for transcripts supported by at least 24 reads, we identified 195 transcripts, of which 111 are predicted to be coding (see Supplementary Data Files 6 and 7 for transcript identity and abundance, respectively). 28 of these were identical to transcripts identified by the exon-level analysis. Strikingly, most of the remainder (130) resulted from small-scale (≤15 nucleotides) differences to our high-confidence exon-level transcripts, demonstrating the complementarity of these approaches. We validated the presence of specific small-scale deletions (3 and 4 amino acid microdeletions, described below) using Sanger sequencing.

### The expression profile of CACNA1C isoforms differs between brain regions

**Fig. 3:**
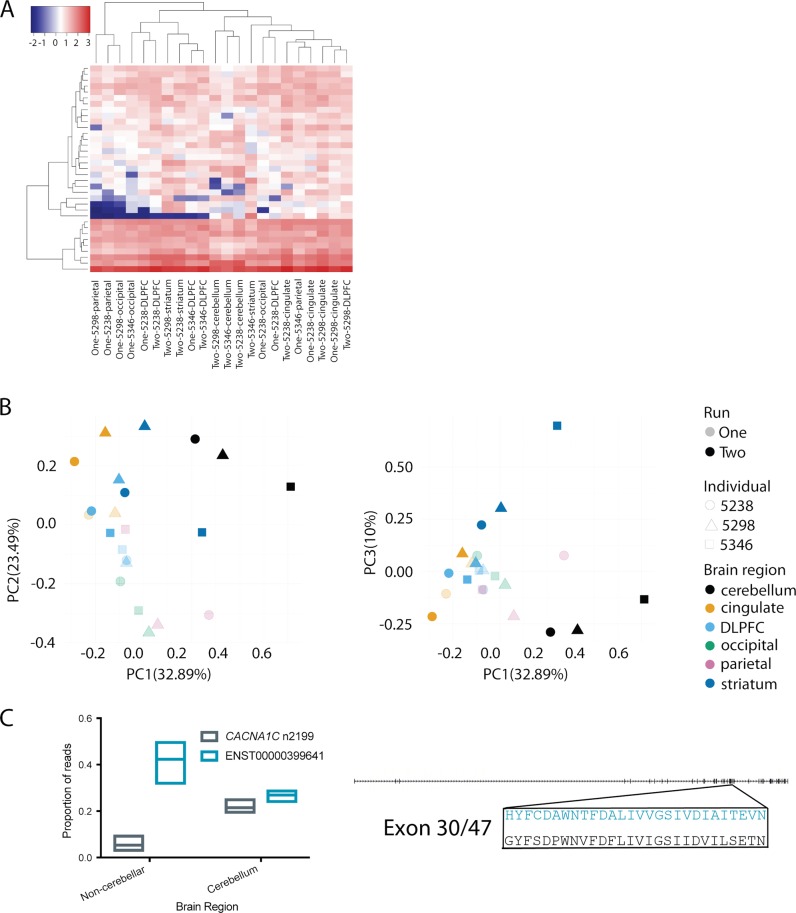
Comparison of CACNA1C isoform expression between individuals and tissues. **a** Transcript expression levels (TPM) across tissues and individuals. “One” and “Two” denote sequencing runs. **b** Principal Component Analysis based on normalised transcript expression. **c** Isoform switching of ENST00000399641 and CACNA1C n2199 in cerebellum. Left panel: box plots show minimum to maximum values with line at mean value. Right panel: the sequences of ENST00000399641 (blue) and CACNA1C n2199 (black) differ in the sequences of their 30th exons. The detail shows the amino acid sequences of this exon in the two transcripts

We examined how *CACNA1C* isoform expression varies across brain regions and between individuals, focussing on transcripts identified using the exon-level approach (similar results were obtained using the splice-site-level transcripts [Supplementary Fig. 4]). We downsampled all libraries to 2729 (the smallest sequencing depth) prior to normalization. Differences between tissues is the main driver of the observed variation in expression between transcripts. Cerebellum and striatum were distinct from the four regions of the cortex (Fig. 3a, b), but expression was similar across individuals, consistent with observations at the transcriptome level [44]. The use of the more permissive filtered set of transcripts for expression estimation (see Methods) further improved the separation between regions, and highlighted potential differences in expression between cortical regions (Supplementary Figs. 5 and 6).

Although we did not find any tissue-specific transcripts amongst our set of high-confidence transcripts, we observed a pronounced transcript expression switch in cerebellum. Outside of the cerebellum *ENST00000399641* was the dominant transcript, whilst in cerebellum, *ENST00000399641* and *CACNA1C n2199* were expressed at similar levels (Fig. 3c).

### Predicted impact of novel isoforms on the Ca_V_1.2 protein model

**Fig. 4:**
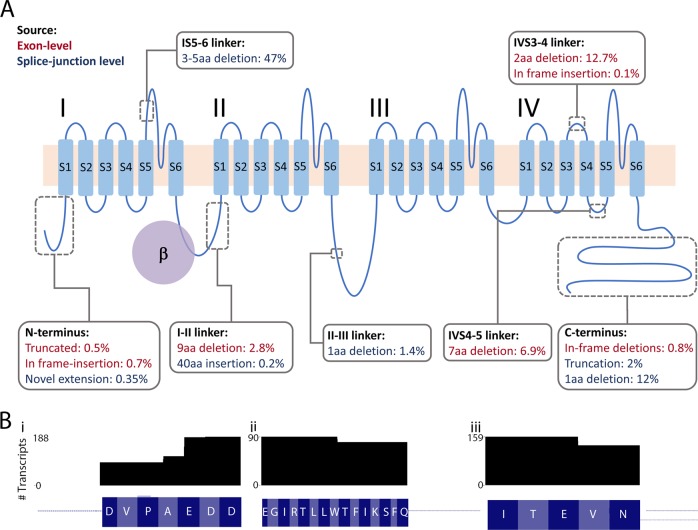
Impact of novel splicing events on the CACNA1C protein model. **a** CACNA1C encodes the primary pore-forming subunit of the Ca_V_1.2. Ca_V_1.2 is formed of four domains (I–IV), each comprised of six transmembrane domains (S1–S6), which are linked by intracellular loops. The obligate beta subunit binds to the I-II intracellular loop, as shown. Grey boxes indicate the location of novel, in-frame insertions and deletions, discussed in the main text. Values indicate the mean proportion of reads containing each variant. Where variants were identified using both analysis approaches, exon-level counts were used to derive abundance (red text); variants identified only using the splice-site-level approach are indicated with blue text. **b** Number of protein isoforms containing three microdeletions: (i) in the I-II linker, (ii) in the IV4–5 linker and (iii) the previously reported microdeletion in the IV3–4 linker

*CACNA1C* encodes the pore-forming Ca_V_1.2 alpha_1_ VGCC subunit. The calcium pore conmprises 24 transmembrane repeats, clustered into 4 domains linked by intracellular loops (Fig. 4a). Among the 83 novel exon-level transcripts we identified, 51 potentially encode functional Ca_V_1.2 channels, whilst the remainder are likely non-coding as they contain deletions in critical membrane-spanning regions and/or frameshifts. Notably, putatively coding isoforms represent 87.8% of total high-confidence reads, demonstrating that while non-coding transcripts are numerous, it is putatively coding transcripts that represent the vast majority of reads (Supplementary Fig. 7).

Around half of putatively coding exon-level transcripts (25 of 51; 26.8% of total coding reads) consist of novel combinations of already annotated exons. The remainder include novel exons and/or deletions (Fig. 3b). Many of the novel splicing events are seen across multiple transcripts. For example, five transcripts (2.8% on average, 5.2% maximally, of total coding reads) predict Ca_V_1.2 protein isoforms with an in-frame deletion in the intracellular I-II linker, and seven (6.9% on average, 11.9% maximally, of total coding reads) contain an in-frame deletion in the IVS4-IVS5 linker (Fig. 4a). Ten protein isoforms (12.7%, on average, 22.3%, maximally, of total coding reads)—including *CACNA1C n1925*, one of the three most abundant transcripts—include a previously described microdeletion in the extracellular IVS3-IVS4 linker (Fig. 4b) [33]. One also contains a 3aa in-frame insertion in this same linker region, highlighting this region as a ‘hotspot’ for alternative splicing, of interest given the relevance of this linker for determining channel properties [33].

Several protein isoforms predict alternative N- and C-termini (Fig. 4a), which are involved in coupling Ca_V_1.2 signalling to intracellular signalling cascades and calcium-dependent inactivation [31, 45]. Variation in these regions is predicted to affect a small fraction of the Ca_V_1.2 pool, but such protein isoforms may still be of biological significance if they show altered inactivation properties and/or coupling to second messenger systems (Fig. 4a).

The splice-site-level analysis identified additional sites of variation (Fig. 4). Most strikingly, 47% of transcripts included an in-frame microdeletion of 3, 4 or 5 amino acids in the IS5–6 linker (corresponding to amino acids D306-E310 in Uniprot entry Q13936). The 4 amino acid microdeletion was previously described in heart but not brain [29], but the 3 and 5 amino acid microdeletions are novel (Fig. 4b). Their functional impact is unknown but the S5-S6 linker is part of the pore-forming region and these microdeletions fall within a region previously implicated in determining channel conductance [46].

## Discussion

We combined long-range RT-PCR with nanopore sequencing in order to characterize the full coding sequence from transcripts of *CACNA1C*. To our knowledge, we are the first to have used this approach successfully in human post-mortem tissue. The vast majority of *CACNA1C* transcripts are novel, and many of these are abundant. We demonstrate marked differences in *CACNA1C* transcript profiles between brain regions, with the cerebellum in particular showing a notable switch in isoform abundance compared with cortex. Our results demonstrate that *CACNA1C* transcripts are much more diverse than previously appreciated, and emphasise the importance of studying full-length isoforms and of access to high-quality human brain tissue.

Despite annotations of very high quality and active curation [19, 47], an increasing number of studies report novel protein-coding sequences and exons in the human genome. The rapid development of long-read sequencing technologies opens the unique opportunity to gain an accurate representation of transcript diversity, as each read encompasses a full transcript. This knowledge is particularly critical for genes with complex models, for example a recent study of DSCAM, identified 18,496 transcripts out of over 19,000 of those theoretically possible [48]. We anticipate that long-read sequencing applications will further help decipher transcript expression and splicing, especially for genes expressed in the brain, which exhibits prominent use of alternative splicing and tissue-specific exons [14–18, 49].

Our study highlights the power of long-read sequencing for the annotation and characterisation of alternatively spliced transcripts. We demonstrate a five-fold increase in the number of annotated transcripts for *CACNA1C*, identifying novel exons and deletions within the coding sequence, as well as novel combinations of previously annotated exons. Because of the high quality of the genomic assembly at the *CACNA1C* locus [50], the novel exons, and splice sites we describe are unlikely to result from mapping errors. Instead, our findings support those from transcriptome analyses that indicate that a significant proportion of gene isoforms in human brain remain unannotated [51]. Supporting their potential importance, a number of the novel transcripts are highly abundant individually, and collectively they encompass the majority of reads. Our finding that the vast majority of reads from novel transcripts maintain the *CACNA1C* reading frame also supports the hypothesis that these are functionally relevant and not simply products of “noisy” splicing. Furthermore, we identified several abundant in-frame deletions that are present in a number of transcripts; if translated these could dramatically impact on Ca_V_1.2′s function in the cell [29, 30, 52]. Notably, a number of our novel predicted protein isoforms include alterations in domains known to be important for determining channel properties and coupling to second messenger systems [29, 31, 53], thereby providing testable hypotheses as to their predicted functional impact. Now that the transcript structure of *CACNA1C* is clearer, it will also be of interest to examine the *cumulative* effect of functional variation across the Ca_V_1.2 protein on channel function. Conversely, we identified a relatively low number of annotated transcripts in our dataset. This is perhaps unsurprising, given that many of the currently annotated transcripts are likely predictions from ESTs and incomplete cDNAs. Taken together, these observations demonstrate the importance of long-read sequencing for the accurate characterisation of transcript structure and alternative splicing.

The need to understand the diversity of Ca_V_1.2 isoforms may be of clinical and therapeutic relevance. First, characterization of the complement of full-length *CACNA1C* isoforms is a necessary first step towards understanding how its transcript profile might be altered by a risk variant or disease state. Second, calcium channel blockers, which have Ca_V_1.2 as one of their primary targets, are licensed for cardiovascular indications and have possible utility as a therapeutic strategy for psychiatric disorders [54–57]. Since the Ca_V_1.2 proteins that arise from *CACNA1C* splicing show differential sensitivity to the existing calcium channel blockers [52] it may be possible to selectively target disease-relevant *CACNA1C* isoforms and/or those that are differentially expressed in the brain vs. the periphery, to provide novel psychotropic agents that are both more potent and are freer from peripheral side effects.

Our results indicate differences in the abundance, but not the identity, of *CACNA1C* transcripts between brain regions. Utilising our more conservative exon-level analysis, in most regions there is a single major *CACNA1C* isoform, with levels of expression almost five-fold higher than the second most highly expressed transcript. However, in the cerebellum there is a switch in transcript abundance, such that the two most abundant transcripts are expressed at similar levels to one another. The consistent nature of this switch in different individuals supports the hypothesis that this is a regulated switch in expression. In contrast, our analysis demonstrated only minor differences in the transcript profile between individuals (Fig. 3b). Given these findings, we are therefore confident that our analysis is likely to have identified the most common and abundant *CACNA1C* transcripts present in adult human brain. However, we anticipate that the relative abundance of these transcripts will likely differ between individuals. Our splice site-level analysis found broadly the same results, although there was no longer a single dominant isoform in each region, largely due to variation in the IS5–6 linker. While we have validated these variations, increased certainty in their abundance and impact on overall transcript structure will likely require further improvements in exon boundary identification with nanopore sequencing. The details of the *CACNA1C* transcript structure revealed here will facilitate the mining of existing large-scale RNA-Seq datasets to investigate these differences between individuals (and between subgroups based on e.g. genotype or disease status).

In summary, our findings demonstrate the utility of long-range amplicon sequencing for the identification and characterisation of gene isoform profiles. More specifically, they demonstrate that the human brain *CACNA1C* transcriptional profile is substantially more complex than currently appreciated. Our approach focused on annotated 5′ and 3′ ends; thus, there is a need for further in depth investigation, e.g. using RACE and capture sequencing. Understanding the functional consequences of this isoform diversity will advance our understanding of the role of VGCCs in the human brain and their involvement in psychiatric disorders [2–4]. Finally, some of the novel isoforms we present here may prove novel and selective therapeutic targets for these disorders [54].

## Supplementary information

Supplementary Information

Dataset 1

Dataset 2

Dataset 3

Dataset 4

Dataset 5

Dataset 6

Dataset 7

## Data Availability

Nanopore sequencing reads generated and analysed in the present study are available from ENA (*PRJEB34660*). Transcript isoforms are available as supplementary data and can be direct visualised and downloaded via our UCSC Genome Browser Track Hub (https://genome-euro.ucsc.edu/cgi-bin/hgTracks?hubUrl=https://opendata.earlham.ac.uk/opendata/data/CACNA1C_isoforms/hub.txt&genome=hg38).
