# Supplementary material for: Long-read sequencing reveals the complex splicing profile of the psychiatric risk gene CACNA1C in human brain
Source: Mol Psychiatry. 2019 Nov 6;25(1):37–47. doi: 10.1038/s41380-019-0583-1 (PMC6906184; doi:10.1038/s41380-019-0583-1)
Supplement: Supplementary file 1 — Supplementary Information [file 41380_2019_583_MOESM1_ESM.docx]

**Supplementary Information**

**Supplementary Methods**

All molecular biology protocols were conducted according to manufacturer’s recommendations unless specifically noted otherwise.

***RNA extraction and RT-PCR***

RNA was extracted from cerebellum, striatum, dorsolateral prefrontal cortex [DLPFC], cingulate cortex, occipital cortex and parietal cortex. Tissue was disrupted with the TissueLyser LT (Qiagen, UK) using Rnase-free 5mm stainless steel beads in QIAzol Lysis Reagent (Qiagen). RNA was extracted using the Qiagen RNeasy Lipid Tissue Mini Kit (Qiagen, UK) and eluted in RNase-free water. RNA concentration and RNA integrity (RNA Integrity Number equivalent; RINe) were measured using a Tapestation 2200 (Agilent, UK). All samples had a RINe of >7.0. RNA (1µg per sample) was converted into cDNA with GoScript^TM^ Reverse Transcriptase (Promega, UK) using oligoDT priming.

Full-length CDS amplicons of *CACNA1C* were obtained by PCR using primers located 5’ to the translation start site in Exon 1B and 3’ to the stop codon in the 3’ untranslated region. Specifically, *CACNA1C* was amplified from cDNA equating to 125ng RNA template from the eighteen human brain samples using PrimeSTAR GXL DNA Polymerase (Takara). Tailed primers were designed to amplify the ~6.5kb *CACNA1C* coding sequence and included sequence to allow further amplification and barcoding. Forward and Reverse primers were tttctgttggtgctgatattgcCATTTCTTCCTCTTCGTGGCTGC and acttgcctgtcgctctatcttcCCAGGTCACGAGAACAGTGAGG, respectively (*CACNA1C* sequence in capital letters). Amplification was conducted for 25 cycles of 98^o^C for 10 sec; 57^o^C for 15 sec; 68^o^C for 7 min.  PCR products were separated on a 1.5% agarose gel (visualised with GelGreen, Biotium, UK). Full-length CDS (~6.5kb) products were excised and purified using the Qiagen Gel Purification Kit (Qiagen, UK). DNA was barcoded using the Oxford Nanopore Technologies (ONT) PCR Barcoding Kit (EXP-PBC001) using PrimeSTAR GXL DNA Polymerase and 12 cycles of PCR amplification, as described above. PCR products were purified with the Qiagen PCR Purification Kit (Qiagen, UK) and quantified by Qubit (ThermoFisher, UK).

***Nanopore Sequencing of the full-length CACNA1C CDS***

The PCR Barcoding Kit (EXP-PBC001, ONT) contains unique twelve barcodes, hence the 18 samples were split across two flowcells with 12 samples run on each. Six samples were sequenced on both flowcells to allow comparison and normalisation between sequencing runs. Samples and barcodes used in each sequencing run are described in Supplementary Table 4.

For each sequencing run, 132ng of each of the twelve samples was pooled and re-purified using 0.4x Agencourt Ampure XP beads (Beckman Coulter, UK) to concentrate the sample and remove any contaminating small DNA products. The presence of full-length DNA product in the pool was confirmed by 2200 or 4200 TapeStation (Agilent) analysis using gDNA screentape (Supplementary Figure 8) and the sample measured again by Qubit to ensure the presence of sufficient product for nanopore library preparation.

Sequencing libraries were prepared with the 2D Nanopore Sequencing Kit (SQK-LSK208, ONT) using 1µg of *CACNA1C* DNA. DNA end-repair and dA-tailing was performed using the NEBNext Ultra II End-Repair/dA-tailing Module (NEB, UK) in a total reaction volume of 60µl (50µl DNA, nuclease-free water (NFW) and DNA calibration strand (Run1 only), 7 μl Ultra II End-Prep buffer and 3 μl Ultra II End-Prep enzyme) for 5 min at 20ºC and 5 min at 65ºC. Samples were purified using 0.6x Ampure XP beads and eluted in 31µl of NFW. Sample concentrations were measured by Qubit and confirmed retention of >85% of the initial samples. The total volume of *CACNA1C* DNA was utilised for subsequent ligation of sequencing adaptors. Ligation reactions contained 50µl Blunt / TA Ligase Master Mix (NEB), 10 μl Adapter Mix 2D, 2 μl Hairpin Adaptor (HPA), 30µl of DNA and 8µl of NFW and incubations were performed at room temperature. After 10 min incubation 1 μl Hairpin Tether (HPT) was added, the sample mixed and incubated for a further 10 minutes. Adapted DNA was purified with MyOne C1 beads (ThermoFisher). 50μl of beads were washed 2x in 100μl bead binding buffer (BBB) and resuspended in 100μl BBB before use. Beads were mixed 1:1 with adapted DNA, incubated on a rotor for 5 min at room temperature to bind DNA to the beads and collected using a magnetic stand. Bead-bound DNA was washed 2x with 150μl of BBB on the magnetic stand and left briefly to dry the beads. Beads were resuspended in 15μl of elution buffer (ELB) and incubated at 37ºC for 10 min to elute DNA from the beads. After pelleting the beads on the magnetic stand, 14μl of supernatant containing the adapted DNA library in ELB was collected. Library concentrations were measured by Qubit, recovering >25% of the original starting amount.

Flowcells were primed with a mix of 480 μl Running Buffer with Fuel Mix (RBF) and 520 μl NFW as per manufacturer's instructions. A loading mix of 35 μl RBF, 25.5 μl library loading beads (LLB), 12 μl *CACNA1C* library and 2.5 μl NFW was prepared, mixed by pipetting and immediately loaded onto the primed “spot-on” flowcell. Sequencing was performed on fresh flowcells; Run 1 utilised a R9.0 (MIN105) flowcell, while Run 2 utilised a R9.4 (MIN106) flowcell. Pore occupancy was >80% indicating a good library and flowcell. Sequencing was allowed to continue until there was a high probability of >1000 high-quality (2D pass) reads from each barcoded sample. Run 1 was base-called with the Epi2Me cloud based service (2D Basecalling plus Barcoding for FLO-MIN105 250bps - v1.125). Run 2 was base-called with Albacore V1.1.0 (FLO-MIN106, SQK-LSK208, barcoding) after the withdrawal of the Epi2Me base-calling service. The absence of a base-calling model for R9.0 in the Albacore software prevents the base-calling of Run1 with this software. Sequencing library metrics (Supplementary Table 5) were generated by Poretools [(Loman and Quinlan 2014)](https://paperpile.com/c/K8DwLs/sVYu) and PycoQC [(Leger and Leonardi 2018)](https://paperpile.com/c/K8DwLs/pXsh).

***Validation of novel exons and junctions***

In the case of novel exons, two sets of nested PCRs were conducted: one spanning the upstream exon and the novel exon sequence (‘5’ confirmation’ in Supplementary Table 2), and a second spanning the novel exon sequence and the downstream exon (‘3’ confirmation’ in Supplementary Table 2). Novel junctions were confirmed using a single round of PCR using one primer spanning the novel junction, with the second primer located in the neighboring exon. PCR reactions were performed using iIlustra PuReTaq Ready-To-Go PCR beads (GE Healthcare Life Sciences, UK). PCR reactions were cycled as follows: 95^o^C for 5 min; 35 cycles of 95^o^C for 35 sec, 30 sec at annealing temperature (see Supplementary Table 2); 72^o^C for 2 min, followed by a final 5 min extension at 72^o^C. Primers sequences and conditions are shown in Supplementary Table 2. PCR products were separated on agarose gels. Products of the predicted size were excised, cleaned using the Qiagen Gel Extraction Kit and ligated into pGEM-T Easy vector (Promega) for Sanger sequencing.

***Impact of RNA quality on amplification of full-length CACNA1C CDSs***

Eleven brain RNA samples (see supplementary table 3) with an average RIN of 8 were pooled and aliquoted into eight samples of 7 μl (875ng each at 125 ng/μl). Each aliquot was heated at 72ºC for different periods of time (0, 2, 5, 10, 20, 35, 60 or 90 minutes) to degrade the RNA. The RINe for each aliquot was then assayed using the Agilent 4200 TapeStation system.

Reverse transcription used 500 ng of RNA and was performed using GoScript^TM^ Reverse Transcriptase. *CACNA1C* amplification was performed as described above, using 5μl of cDNA and 30 cycles of amplification. Forward and Reverse primers were: CATTTCTTCCTCTTCGTGGCTGC and CCAGGTCACGAGAACAGTGAGG, respectively, i.e. identical to those used in the main experiment, but lacking the barcoding tails. Successful amplification of PCR product was confirmed and visualised using both agarose gel electrophoresis and 4200 Tapestation analysis using equal proportions of each PCR. Samples from three additional adult donors were utilised specifically for this experiment to provide the required range of high quality and partially degraded samples.

**Supplementary Table 1: Demographics of tissue donors**

| Case ID | Age (years) | Sex | PMI | Ethnicity | Brain RIN | Cause of death |
| --- | --- | --- | --- | --- | --- | --- |
| 5238 | 37.2 | M | 10.5 | AA | 8.9 | Multiple blunt force injuries |
| 5298 | 50.3 | M | 12.5 | AA | 8.7 | Multiple injuries |
| 5346 | 25.1 | F | 24.5 | AS | 8.3 | Multiple injuries |

PMI: postmortem interval; RIN: RNA Integrity Number; M: male; F: female; AA: African American; AS: Asian

**Supplementary Table 2: primers and PCR conditions used to confirm novel exons and junctions.**

| Novel exon/junction | | Forward primer | | Reverse primer | | Annealing temperature |
| --- | --- | --- | --- | --- | --- | --- |
| Novel exon A | 5’ confirmation (round 1) | Exon 1B F2 | GGTCAATGAGAATACGAGGATG | Exon A R2 | AGCCTCGTGTCATTCTGCT | 53 |
|  | 5’ confirmation (round 2) | Exon 1B F | GAATCAGGTAATCGTCGGCGG | Exon A R | TGAAGACAGCATCTGCGTC | 53 |
|  | 3’ confirmation (round 1) | Exon A F2 | CCTCATCCTGGTCCCCAGC | Exon 2 R2 | CTGCCCATCAGCTTAGCCTG | 53 |
|  | 3’ confirmation (round 2) | Exon A F | AGCAGAATGACACGAGGCT | Exon 2 R | AGCTGACTGTGGAGATGGTC | 53 |
| Novel Exon B | 5’ confirmation (round 1) | Exon 3 F2 | ACGCCACCAATTCCAACCTG | Exon B R2 | TCTCCAATCTGGGATGTTCCTC | 50 |
|  | 5’ confirmation (round 2) | Exon 3 F | TTGCCAATTGTGTGGCCTTAG | Exon B R | CTACTCTAGTTGGGCTGAGTT | 50 |
|  | 3’ confirmation (round 1) | Exon B F2 | AACTCAGCCCAACTAGAGTAG | Exon 4 R2 | GGTGAAAGAGGAGTCCATAGG | 50 |
|  | 3’ confirmation (round 2) | Exon B F | GAGGAACATCCCAGATTGGAGA | Exon 4 R | TCTAGTAGGTTCCAGCCGTTG | 50 |
| Novel Exon D | 5’ confirmation (round 1) | Exon 7 F2 | CAGTGCATCACCATGGAGG | Exon D R2 | TCTGCCTCAAGAGGAATCACTCT | 53 |
|  | 5’ confirmation (round 2) | Exon 7 F | GCACGGCATCACCAACTT | Exon D R | CTCCTGTGACCGAAGGGGAC | 53 |
|  | 3’ confirmation (round 1) | Exon D F2 | GTCCCCTTCGGTCACAGGAG | Exon 8 R2 | CAGGGTAACTCATAGCCCATAGC | 53 |
|  | 3’ confirmation (round 2) | Exon D F | AGAGTGATTCCTCTTGAGGCAGA | Exon 8 R | CGCTCAACACACCGAGAACCA | 53 |
| Novel Exon 9-13 junction |  | Exon junction 9.13 F | CCCGAAACAACACGGCAAACA | Exon 13 R | ACACACGACGAAGCAGTCAAA | 55 |
| Novel Exon 14-23 junction |  | Exon 14 F2 | CATCCTTGCTGAACTCTGTGC | Exon junction 14.23 R | AGCAGTCATCTGAAACACAGTGA | 53 |
| Novel Exon 37-44 junction |  | Exon 37 F | TGAAACACCCTGTGGTAGCAG | Exon 37.44 R | GCCCTCAGGAAGGCACAGA | 55 |

**Supplementary Table 3: Demographics of additional tissue donors used for RNA degradation study**

| Case ID | Age (years) | Sex | PMI | Ethnicity | Brain RIN | Cause of death |
| --- | --- | --- | --- | --- | --- | --- |
| 5244 | 21.2 | M | 28 | AA | 8.7 | Multiple injuries |
| 5579 | 41.9 | F | 13 | CAUC | 6.3 | Multiple injuries |
| 5717 | 51.9 | M | 35.5 | CAUC | 8.9 | Sarcoidosis |

PMI: postmortem interval; RIN: RNA Integrity Number; M: male; F: female; AA: African American; CAUC: Caucasian

**Supplementary table 4: Samples and pass reads in each sequencing run**

| Sample | Barcode | Run | Run1 reads | Run2 reads |
| --- | --- | --- | --- | --- |
| 5238 cingulate | BC001 | 1, 2 | 4836 | 6752 |
| 5238 DLPFC | BC002 | 1, 2 | 7227 | 10484 |
| 5238 occipital | BC003 | 1 | 4630 |  |
| 5238 parietal | BC004 | 1 | 6024 |  |
| 5238 cerebellum | BC003 | 2 |  | 3735 |
| 5238 striatum | BC004 | 2 |  | 8778 |
| 5298 cingulate | BC005 | 1, 2 | 6287 | 9329 |
| 5298 DLPFC | BC006 | 1, 2 | 5660 | 8849 |
| 5298 occipital | BC007 | 1 | 2729 |  |
| 5298 parietal | BC008 | 1 | 2729 |  |
| 5298 cerebellum | BC007 | 2 |  | 6725 |
| 5298 striatum | BC008 | 2 |  | 8116 |
| 5246 cingulate | BC009 | 1, 2 | 1760 | 2421 |
| 5346 DLPFC | BC010 | 1, 2 | 2572 | 3403 |
| 5346 occipital | BC011 | 1 | 4859 |  |
| 5346 parietal | BC012 | 1 | 3681 |  |
| 5346 cerebellum | BC011 | 2 |  | 7609 |
| 5346 striatum | BC012 | 2 |  | 4130 |

**Supplementary table 5: Sequencing metrics for each nanopore sequencing run**

|  | Run1 | Run2 |
| --- | --- | --- |
| Flowcell | R9.0 (MIN105) | R9.4 (MIN106) |
| Basecalling | Epi2Me v1.125 | Albacore V1.1.0 |
| Total reads | 112024 | 126314 |
| Pass, barcoded reads | 52994 | 80331 |
| Yield (Mb) | 315 | 480 |
| Median length (pass, barcoded reads only) | 6363 | 6451 |
| Median Q-score (pass reads only) | 12.75 | 15.89 |
| DNA calibration strand added | Y | N |

**Supplementary Figure 1**


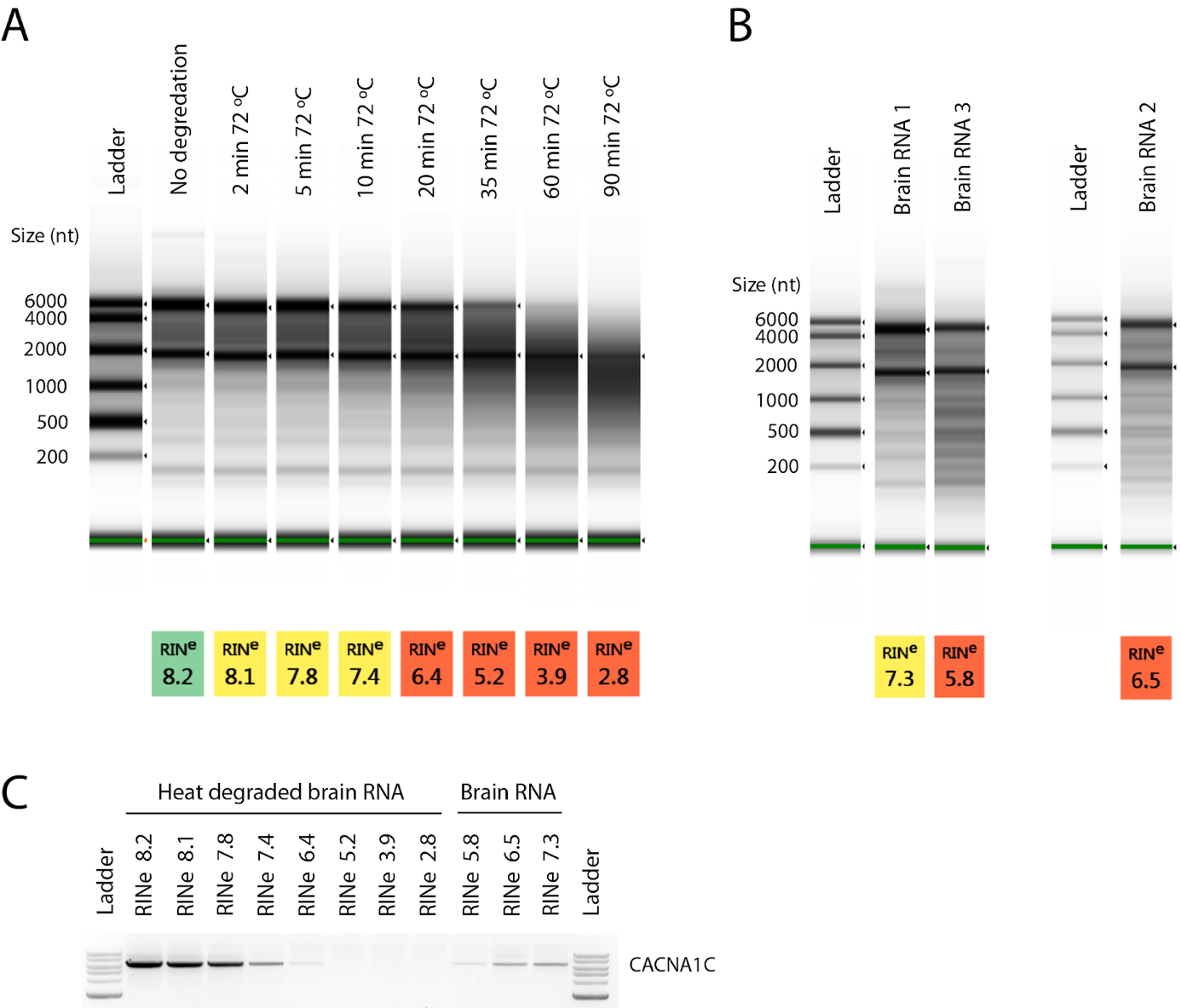


Supplementary Figure 1: High-quality RNA is required for amplification of *CACNA1C* CDS. The quality of RNA from post-mortem or tissues samples can be highly variable with many samples having undergone significant degradation. Conversely, RT-PCR and sequencing of long and/or full-length cDNAs requires the sample RNA to be of sufficient integrity to contain undegraded transcripts. To investigate how RNA quality impacts the feasibility of amplifying and sequencing full-length genes, and to establish a minimum recommended quality value, we artificially degraded brain RNA to create a series of RINe values and investigated the effect of RNA quality on full-length CACNA1C CDS amplification. RNA quality varied from RINe 8.2 (undegraded RNA) to 2.8 (90 min at 72ºC) (Figure S1A. *CACNA1C* amplified strongly in samples with a RINe of ~8, with lower quality samples showing decreasing amounts of *CACNA1C* and no product from samples with a RINe of ~<5 (Figure S1C). As RNA degradation from heat treatment may not accurately reflect standard sample degradation, we attempted *CACNA1C* amplification in three untreated striatal samples with RINe of 7.3, 6.5 and 5.8 with similar results (Figure S1B,C). These results suggest a minimum RINe value of 6 for generating long amplicons from post-mortem brain samples and a recommended RINe of >7 for robust amplification. (**A**) Tapestation profile and RINe quality values of brain RNA after heat treatment at 72ºC. Untreated RNA had a RINe of 8.2. (**B**) Tapestation profiles and RINe quality values of untreated striatal brain RNA samples. Only lanes of interest from Tapestation profiles are shown. (**C**) Agarose gel showing amplification of the ~6.5kb *CACNA1C* CDS from RNA of varying quality.

**Supplementary Figure 2: Mapping pipeline for the annotation of novel exons and novel transcripts**


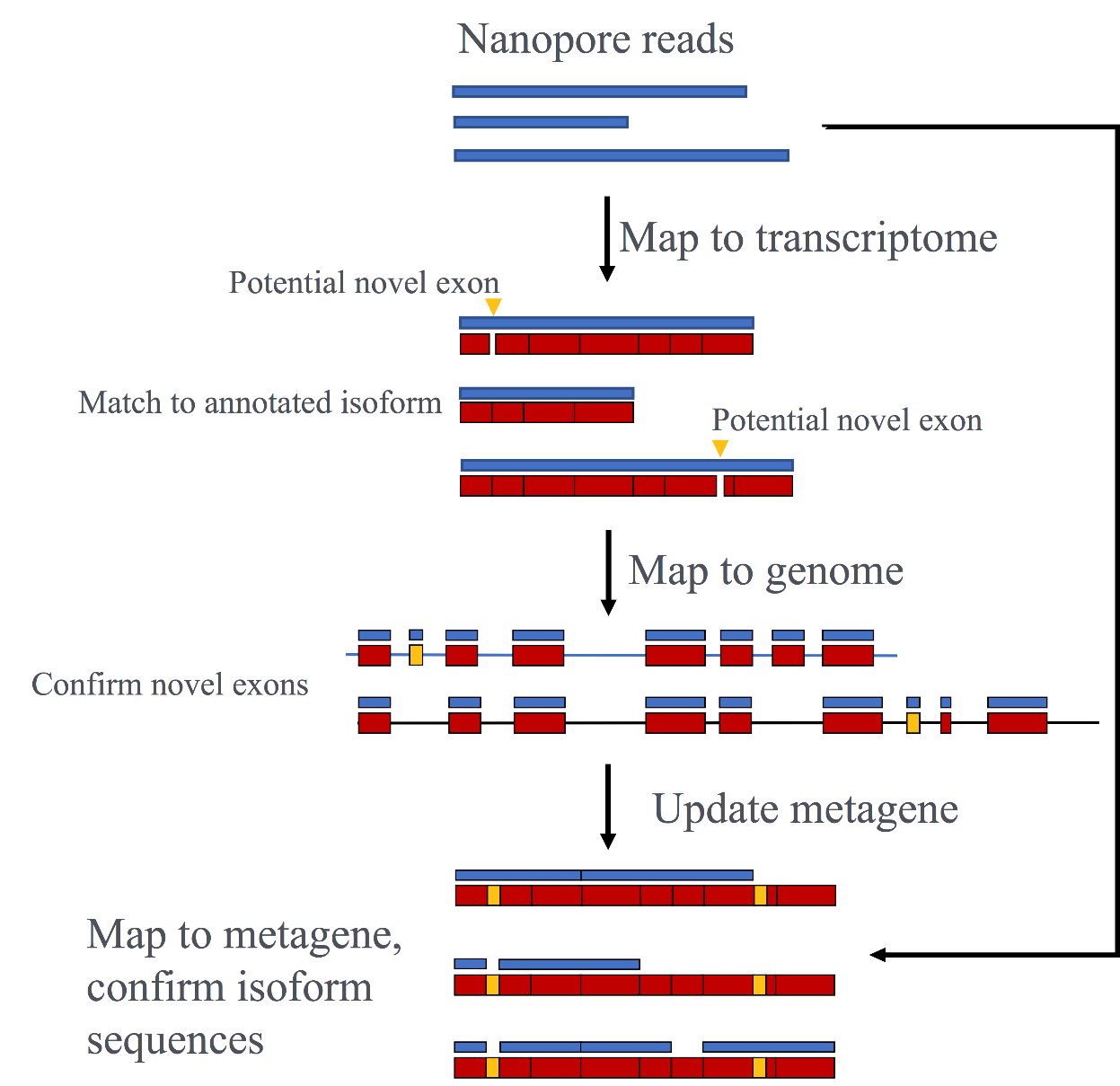


**Supplementary Figure 3: High confidence *CACNA1C* isoforms identified by Nanopore sequencing**

**
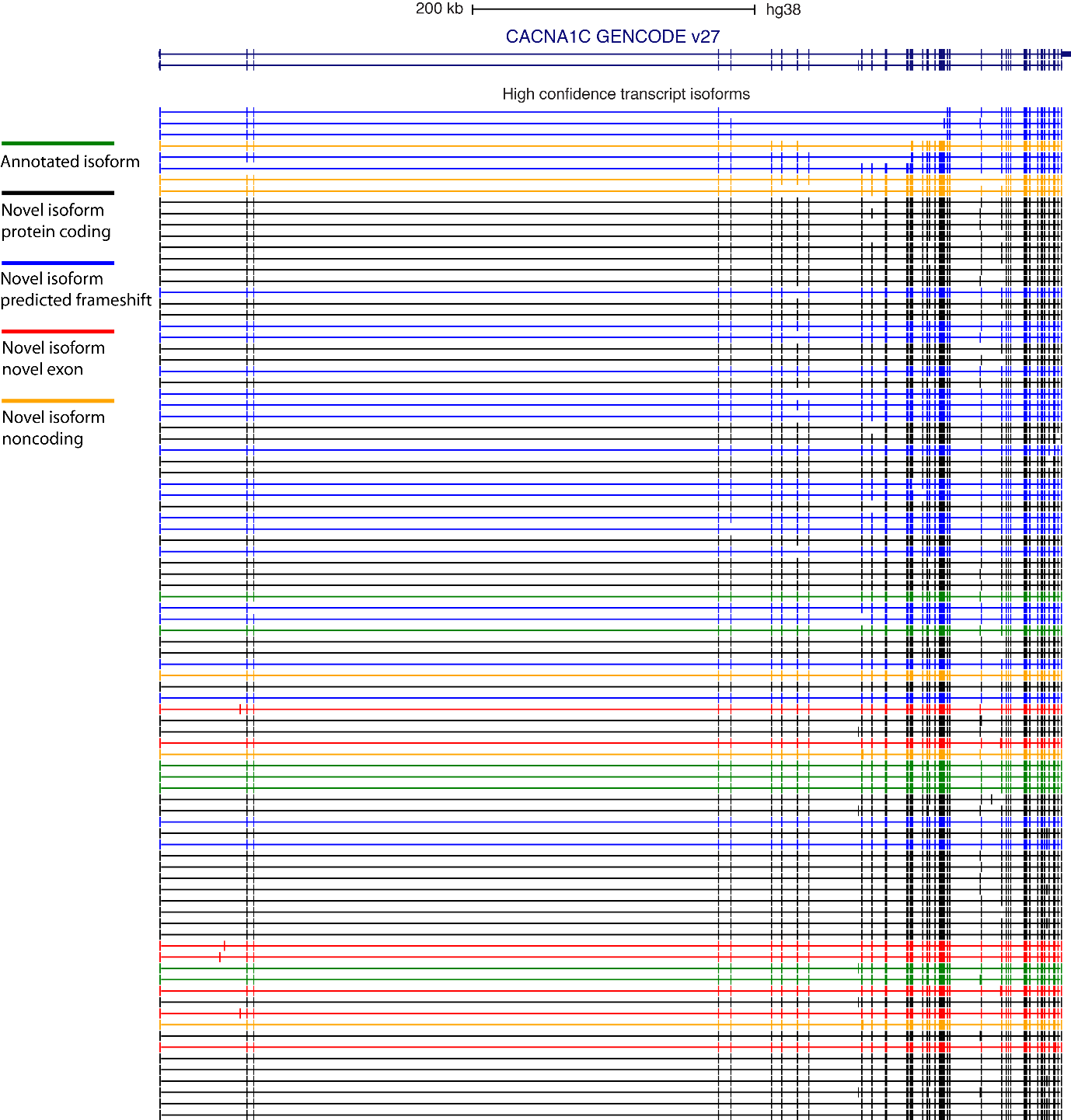
**

USCS genome browser image of all high confidence CDS transcripts from the exon-level analysis. Representative annotated transcripts from GENCODE v27 shown. Colours of nanopore identified transcripts represent different transcript classes based on their coding potential. Two novel exon transcripts (red) are further annotated as noncoding, the remainder as coding.

**Supplementary Figure 4**


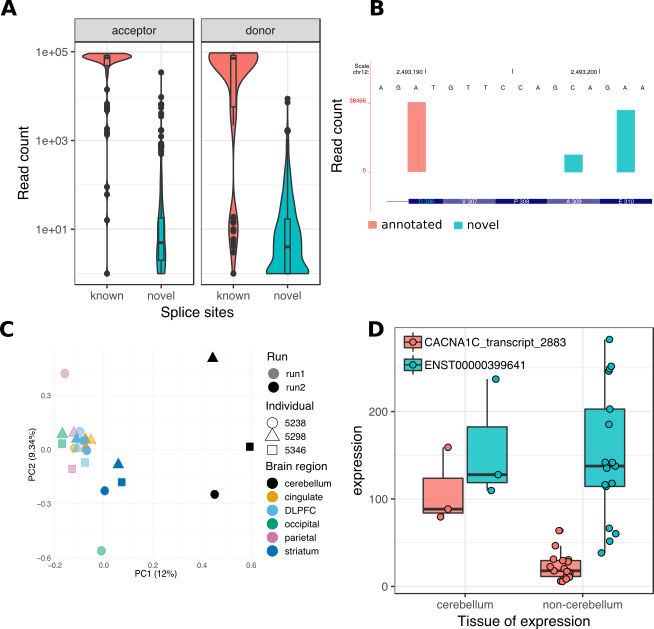


(A) Comparison of annotated and novel splice sites read coverage. (B) Identification of highly supported novel splice sites. (C) Principal Component Analysis based on normalised transcript expression after integration of updated splice site annotation. (D) Isoform switching of *ENST00000399641* and *CACNA1C n2883* (known as n2199 in the exon-level analysis) in cerebellum.

**Supplementary Figure 5: Clustering of *CACNA1C* isoform expression between individuals and tissues with the permissive set of filtered transcripts**
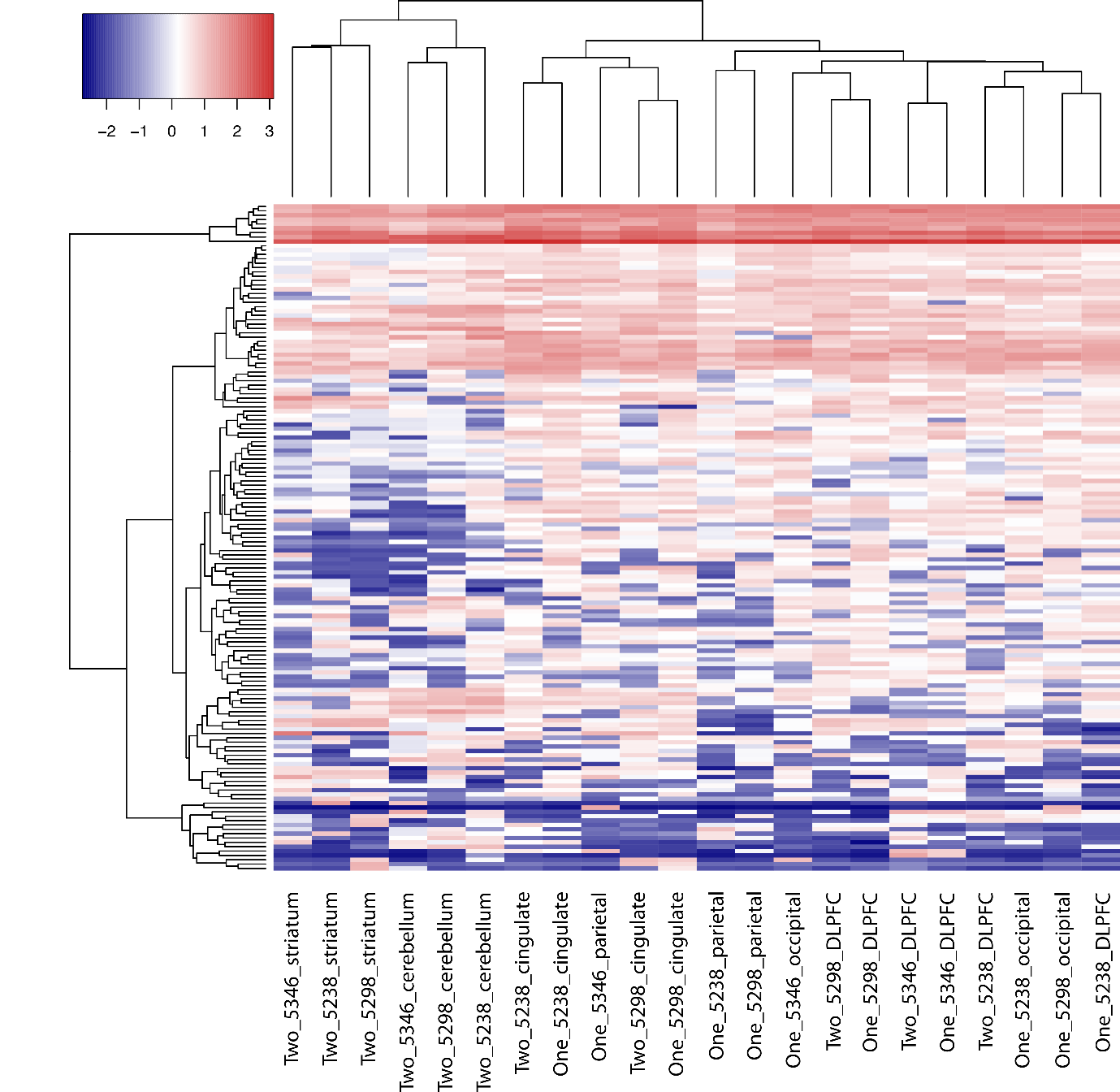


Transcript expression levels across tissues and individuals. “One” and “Two” denote sequencing runs. Due to the large difference in sequencing depth between samples, all libraries were downsampled to the smallest sequencing depth. Inclusion in “permissive” transcript set required isoform identification in at least 2 libraries with a minimum of 24 reads in total. Permissive set of isoforms demonstrates improved separation between brain regions.

**Supplementary Figure 6: Principal Component Analysis of *CACNA1C* isoform expression between individuals and tissues with the permissive set of filtered transcripts**


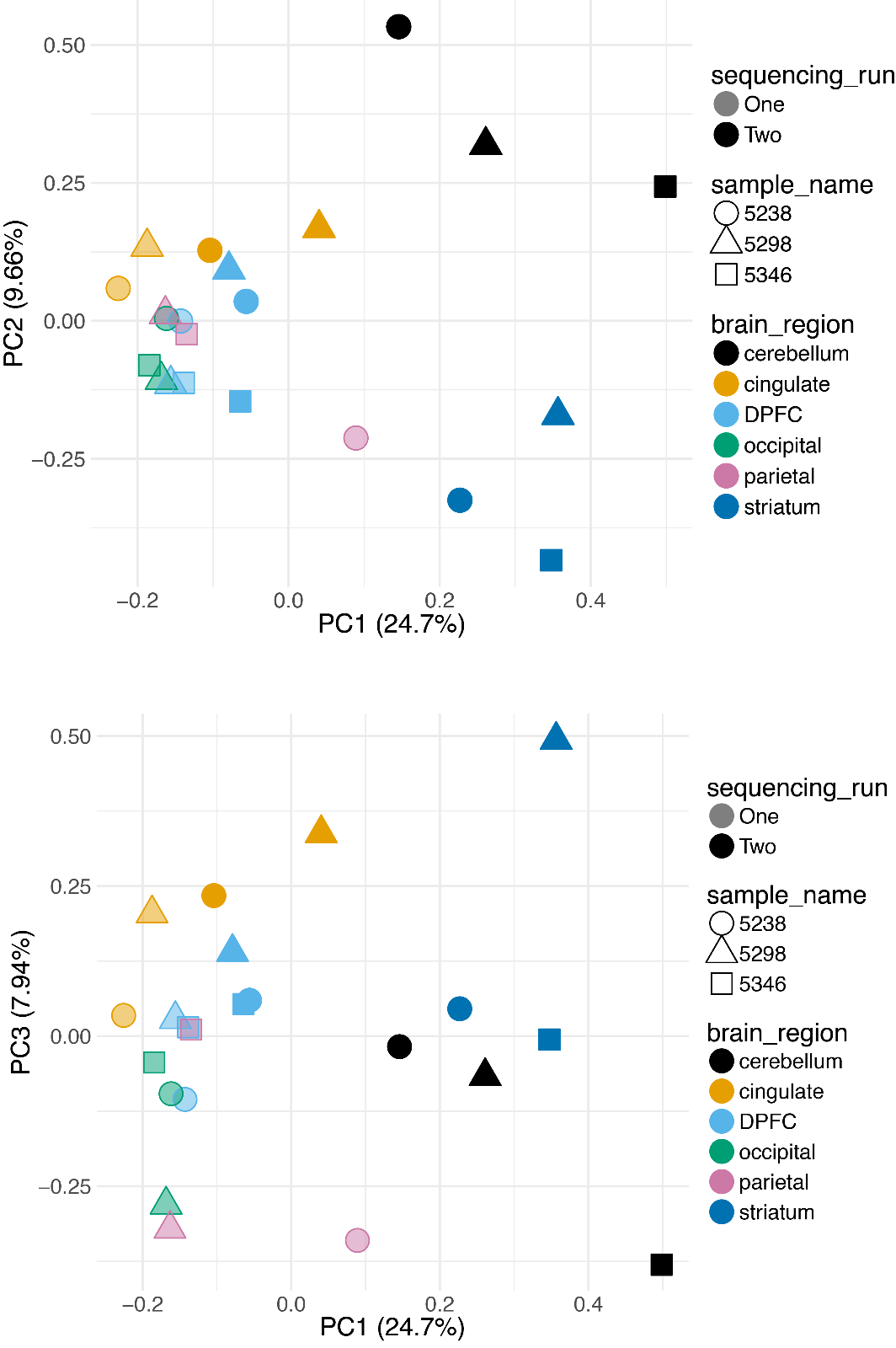


Principal Component Analysis based on normalised transcript expression. Due to the large difference in sequencing depth between samples, all libraries were downsampled to the smallest sequencing depth. Inclusion in “permissive” isoform set required transcript identification in at least 2 libraries with a minimum of 24 reads in total. Permissive set of transcripts demonstrates improved separation between brain regions.

**Supplementary Figure 7**


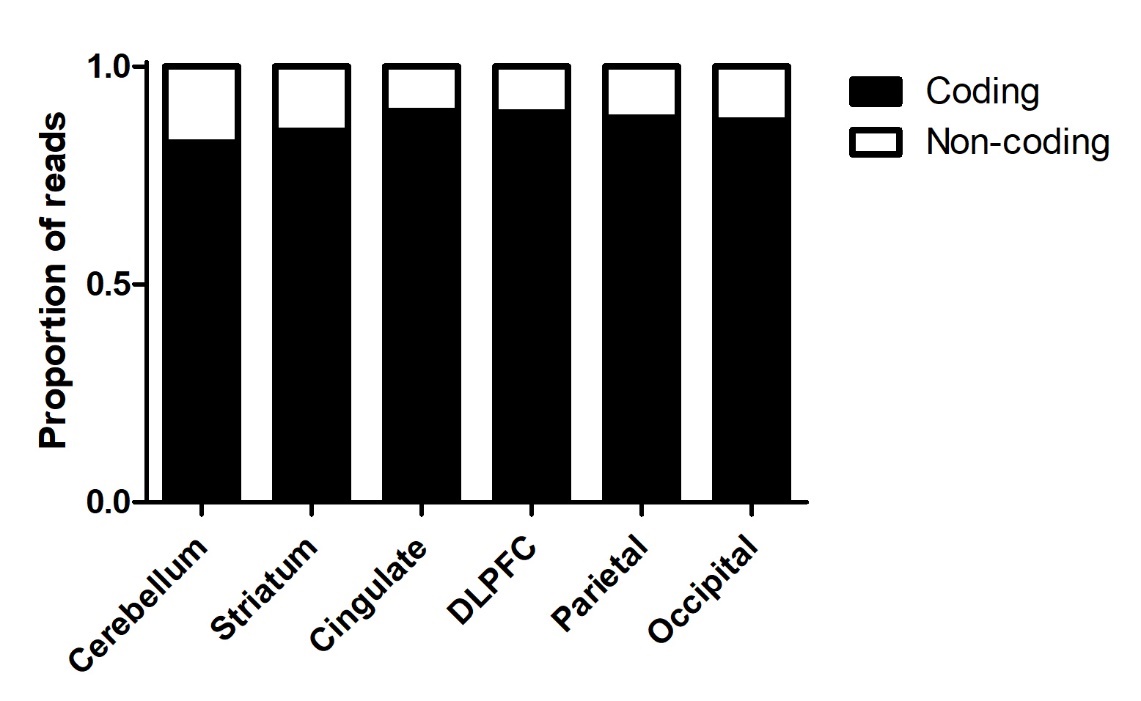


The majority of reads map to *CACNA1C* transcripts that predicted to encode a functional protein. The figure shows the proportion of *CACNA1C* reads that are predicted to encode a functional protein (black bars), compared to those which introduce a frame-shift or which include sequence variants predicted to disrupt transmembrane regions (‘non-coding; white bars) in the different brain regions studied.

**Supplementary Figure 8: Full-length, highly pure pooled CACNA1C amplicons.**

A) Run1 B) Run2. Slight apparent shift of amplicon to a larger size is due to slight inaccuracy inherent in gDNA screentape where there is only a lower marker for sizing.


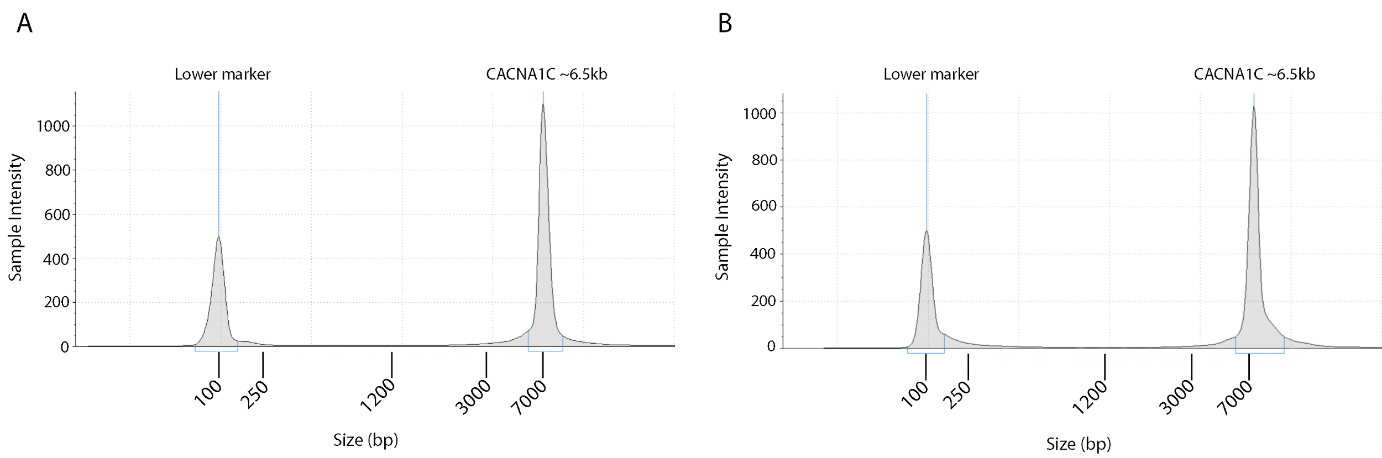


**Supplementary Data Files:**

*Supplementary Data 1:*

Details of novel exons in .bed format.

*Supplementary Data 2:*

Identity of low confidence transcripts from exon-level analysis in .bed format.

*Supplementary Data 3:*

Abundance of low confidence transcripts from exon-level analysis in different samples.

*Supplementary Data 4:*

Identity of high confidence transcripts from exon-level analysis in .bed format.

*Supplementary data 5:*

Abundance of high confidence transcripts from exon-level analysis in different samples.

*Supplementary Data 6:*

Identity of splice-site analysis transcripts in .bed format.

*Supplementary Data 7:*

Abundance of splice-site analysis transcripts from exon-level analysis in different samples.
